# Effectiveness of diabetes self-management education via a smartphone application in insulin treated type 2 diabetes patients – design of a randomised controlled trial (‘TRIGGER study’)

**DOI:** 10.1186/s12902-018-0304-9

**Published:** 2018-10-22

**Authors:** Anne Meike Boels, Guy Rutten, Nicolaas Zuithoff, Ardine de Wit, Rimke Vos

**Affiliations:** 1 Julius Center for Health Sciences and Primary Care, University Medical Center Utrecht, Heidelberglaan 100, 3584 CX Utrecht, The Netherlands; 2 Centre for Nutrition, Prevention and Healthcare, National Institute of Public Health and the Environment, PO Box 1, 3720 BA Bilthoven, The Netherlands; 3 Leiden University Medical Center, Dept Public Health and Primary Care, LUMC-Campus, Turfmarkt 99, 2511 DP The Hague, the Netherlands

**Keywords:** Type 2 diabetes mellitus, Insulin therapy, mHealth, eHealth, Self-management, Behavioural change, Triggers, Cost-effectiveness, Diabetes education, Hypoglycaemia

## Abstract

**Background:**

Health care providers aim to stimulate self-management in type 2 diabetes (T2DM) patients. However, they have a limited number of patient contacts to do this. With the growing number of T2DM patients, innovative and cost-effective interventions to promote self-management are needed. We aim to evaluate the effectiveness of diabetes self-management education via a smartphone app in T2DM patients on insulin therapy.

**Methods:**

Non-blinded two-arm multi-centre randomised controlled superiority trial with parallel-groups and equal randomisation (‘TRIGGER study’). Eligible patients are 40–70 years, on insulin therapy since at least 3 months, with HbA1c > 53 mmol/mol (> 7%). In total 228 patients will be recruited. The intervention group (*n* = 114) will receive diabetes self-management education via a smartphone app to trigger diabetes self-management: unidirectional text messages, free of charge, evidence and psychological theory based, with regard to dietary habits, physical activity, hypoglycaemia and glucose variability. Patients choose their preferred frequency (two to six times per week), topics (two or three additionally to hypoglycaemia, which is an obligatory topic), and duration (6 or 9 months). The control group (*n* = 114) will receive care-as-usual. The primary study endpoint is the HbA1c level after a follow-up of 6 months. The percentage of patients who achieve an HbA1c level ≤ 53 mmol/mol (≤7%) without hypoglycaemia (plasma glucose < 3.5 mmol/L (< 63 mg/dL)) is a co-primary outcome. Secondary outcomes are body mass index, waist circumference, insulin dose, lipid profile, blood pressure, number of hypoglycaemic events, glycaemic variability, self-management (SDSCA), food habits (FFQ), physical activity (IPAQ), health status (EQ-5D-5 L, SF36), diabetes-dependent quality of life (ADDQoL), diabetes treatment satisfaction (DTSQ), satisfaction with the app, the cost-effectiveness of the intervention after 3 months, and sustainability of the intervention effect (3 months extra follow-up in intervention group to compare prolonged to discontinued use of the app). We will use the intention-to-treat principle to analyse data.

**Discussion:**

Innovative solutions are needed to improve the (cost-) effectiveness of self-management for the increasing number of T2DM patients. This trial will provide evidence on the effectiveness of a newly developed smartphone app, designed to trigger diabetes self-management.

**Trial registration:**

Dutch Trial Register NTR5515, registration date: 18 November 2015 (prospectively registered).

**Electronic supplementary material:**

The online version of this article (10.1186/s12902-018-0304-9) contains supplementary material, which is available to authorized users.

## Background

Self-management forms a crucial part of type 2 diabetes (T2DM) treatment, especially for insulin treated individuals. It includes healthy food choices, frequent exercising, regular blood glucose monitoring, and dietary and insulin dose adjustments related to physical activity. These adaptations are necessary to achieve optimal glycaemic control (without much variability and hypoglycaemia) and weight loss, and to prevent both microvascular and macrovascular complications. However, for most patients, diabetes self-management is challenging and difficult to maintain. Besides, results of self-management interventions are often effective in the short-term, but have mixed long-term results [1]. Health care providers aim to stimulate self-management, but due to the limited number of diabetes monitoring visits they are in the position to trigger diabetes self-management only a few times a year [2]. Moreover, with the growing number of T2DM patients and the expected shortage of health care providers in some countries, innovative and (cost-) effective solutions to promote self-management are needed.

A potential low-cost intervention to stimulate diabetes self-management may be the use of a smartphone app. The number of diabetes apps has tremendously increased during the past years. In the iTunes store alone, there was a ten-fold increase from 60 in 2009 [3], to 622 in 2013 [4]. When the number of diabetes apps from Google Play, BlackBerry, Windows and Ovi Store are added to that number, the total number of diabetes apps already exceeded 1800 in 2013 [4]. Unfortunately, many of these diabetes apps have not been studied in medical research, i.e. they are not evidence based. In one study, only 16 out of the 87 identified apps could be found in medical literature; 71 apps were solely found in the iTunes Store [5].

A common feature of a diabetes app is self-monitoring of blood glucose levels, blood pressure, physical activity, weight, medication intake and food intake [3, 5–7]. For blood glucose, blood pressure and physical activity (step counter), automatic data entry is often possible, but apps facilitating these features are likely to be associated with higher costs. Another common feature is interactive communication with health care providers [3, 7]. Many of the apps that have been investigated showed to be effective [6–8]: HbA1c decreased [7, 9, 10] and self-management improved [11]. However, for most patients, daily entry of personal data is a tedious task [6], often resulting in infrequent or non-use of the app [11]. Besides, technologies for automatic data transfer are more expensive, as is true for interactive communication and tailored feedback from health care providers [12].

Another type of a smartphone app, that may have a more sustainable effect on self-management, is an educational app that sends automated app-messages that function as behavioural triggers; it requires less effort from both health care providers and patients, and may be a low-cost solution compared to the abovementioned strategies. We hypothesise that receiving cost free behavioural triggers on preferential topics and with a self-chosen frequency, sent as app-messages via a smartphone app, will result in better diabetes self-management and improved glycaemic control, less weight gain and less glucose variability with less hypoglycaemic events in T2DM patients on insulin therapy.

### Theoretical framework of the intervention

Behavioural triggers (also known as prompts, stimuli or cues to action) play a pivotal role in behaviour change [13]. They play an important role in the ‘Health Belief Model’ [14], in Leventhal’s self-regulation model [15] and in the ‘Transtheoretical Model of Behaviour Change’ [16]. In the ‘Fogg Behavior Model’, triggers are even of paramount importance [13]. In all these theories, behavioural triggers have some overlapping characteristics. Firstly, a trigger stimulates the individual to engage in health-promoting behaviour [14, 15]. Secondly, a trigger can be either internal (i.e. signs and symptoms such as pain or dyspnoea) or external, emerging in various forms: from a warning label on a product to a reminder e-mail from the dentist [14, 15]. Finally, external triggers can have a motivational element (e.g. a trigger that highlights fear), can increase the individuals’ ability to behaviour change, or can function as a signal (e.g. a reminder) [13]. While triggers are import for behavioural change, for a given behaviour to occur more than a single trigger is needed, namely a certain level of perceived health threat, severity, self-efficacy and a sufficient level of motivation [13–16]. The intensity of the trigger will depend on the presence and level of for example motivation (i.e. the less motivated, the more intense the trigger should be to achieve the intended behaviour) [13, 14]. On the other hand, receiving a behavioural trigger can be the last factor to overcome barriers and to adapt the intended behaviour.

Behavioural triggers can be sent as text messages. Evidence on the effectiveness of unidirectional triggers delivered by mobile phone technology (either via app or SMS) so far is mainly based on studies with a small number of patients and a short duration, focussing on feasibility [8, 17–19]. Moreover, studies were conducted in patient samples that may not be representative for Western countries, e.g. in Iraqi patients and in resource-poor patients in the United States (for a greater part low income groups and from ethnic minority groups, frequently without health insurance) [20, 21]. Furthermore, the sustainability of app-triggers or SMS-triggers on diabetes self-management is unknown, but important for successful implementation.

## Methods

### Research questions

We aim to determine the effectiveness of unidirectional evidence based app-messages on self-management of insulin treated T2DM patients. This will be determined via the following research questions (1) What are the effect of the intervention on HbA1c level and on the percentage of patients who achieve an HbA1c level ≤ 53 mmol/mol (≤7%) without hypoglycaemia (plasma glucose < 3.5 mmol/L (< 63 mg/dL) after a follow-up period of 6 months? (2) What are the effects of the intervention on body mass index (BMI), lipid profile, blood pressure, waist circumference, prescribed insulin dose, number of hypoglycaemic events and glucose variability after a follow-up period of 6 months? (3) What are the effects of the intervention on self-management activities, food habits, physical activity, health status, diabetes related quality of life and diabetes treatment satisfaction after a follow-up of 6 months? (4) What is the cost-effectiveness of the app-trigger intervention compared to usual care? and (5) Is the intervention effect sustainable after 3 months of prolonged versus discontinued use of the app in the intervention group?

### Study design

The TRIGGER study is designed as a non-blinded two-arm multi-centre randomised controlled superiority trial with parallel-groups and equal randomisation (1:1). This study protocol is reported following the SPIRIT guideline for standard protocol items in interventional trials [22]. The information on the mHealth intervention is reported following the mHealth evidence reporting and assessment (mERA) checklist [23].

### Ethics and trial registration

The Medical Research Ethics Committee of the University Medical Center Utrecht has approved the study protocol (protocol number: NL53125.041.15, issued October 2015, issue date amendment: May 2016 (reason: additional recruitment in secondary care)). The trial is registered in the Dutch trial register (trial-ID: NTR5515). Important protocol modifications will be communicated with the Medical Research Ethics Committee and the trial register.

### Setting

The study will be conducted in general practices and hospitals across the Netherlands, starting from December 2015 onwards. In the Netherlands, approximately 85% of all T2DM patients are treated in primary care. Practice nurses conduct the regular monitoring visits two to four times yearly. Once a year, the monitoring visit is conducted by, or together with, the general practitioner (GP), but when necessary more often. Patients are referred to hospital based internists/endocrinologists when adequate glycaemic control cannot be achieved or when other problems occur that are beyond the scope of the GP. The internists/endocrinologists closely collaborate with specialised diabetes nurses [2]. All participating sites can be found at the trial website [24]. Participating hospitals are (in alphabetical order): Bethesda Diabetes Research Center, HagaZiekenhuis, Meander Medical Center, Röpcke-Zweers Ziekenhuis and Ziekenhuis Gelderse Vallei.

### Participants

Eligible participants are T2DM patients aged 40–70 years, treated for their diabetes by the recruiting health care provider, sufficiently fluent in Dutch, on insulin treatment since at least three months, and with HbA1c > 53 mmol/mol (> 7%) at most recent measurement. Logistic requirements are the possession of an email address and a smartphone. In the Netherlands, 96% of the population has access to a personal computer [25]. In 2016, 89.6% of the population between 45 and 65 years old had access to a mobile phone with internet capacity, while among those above 65 years only 50.9% had access [26]. We used age as an inclusion criterion because we expect the probability for patients > 70 years to possess a smartphone to be lower than in the younger patient group and besides, for many patients aged > 70 years a less stringent glycaemic target is recommended according to the Dutch Primary Care Guidelines [2]. Patients are excluded when they have a history of alcoholism, drug abuse, dementia or a major psychiatric disorder that is likely to invalidate informed consent or limit the ability of the individual to comply with the protocol requirements.

### Recruitment

**Fig. 1:**
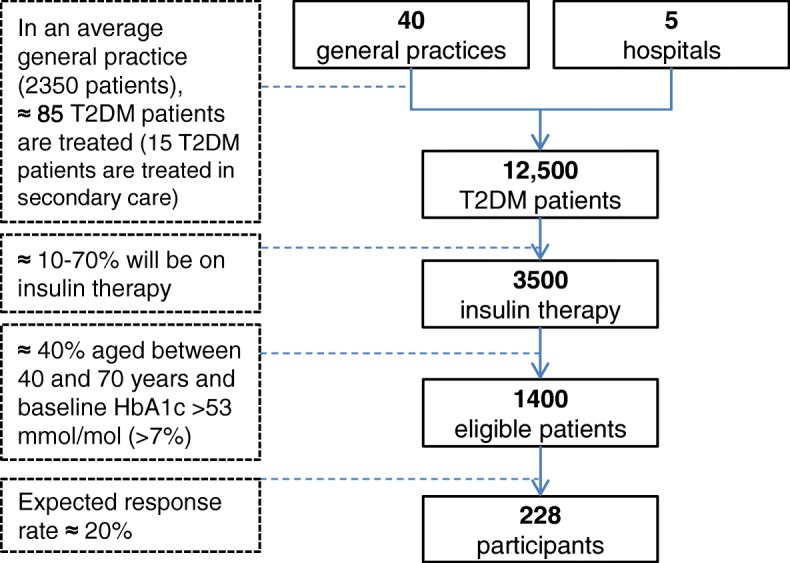
Participant flowchart with the expected number of patients

Most general practices are recruited via diabetes care groups. These care groups organise and coordinate the diabetes care programme, and are responsible for the delivery of diabetes care [27]. Care groups in the Netherlands are similar to Accountable Care Organizations in the USA and Clinical Commission Groups in the UK [27]. We expect that about 40 general practices and five small to medium sized hospitals are needed to enrol the required number of 228 participants (see Fig. 1 and ‘Sample size’ below). When a general practice or a hospital consents to participate, the researchers will instruct the practice nurse or diabetes nurse. Afterwards the practice nurse, diabetes nurse, GP or hospital data manager selects the patients based on a query in the electronic medical record, the practice nurse or the diabetes nurse conducts the informed consent procedure and performs the data collection.

### Randomisation

Simple, fixed block randomisation will be generated at the patient level. Randomisation will be performed centrally using a web-based computerised random-number generator provided by an independent contractor (Research Online, https://random.mendixcloud.com/login.html) at the research centre. For general practices, the coordinating researcher will generate the allocation sequence, in the order in which patients are enrolled, and forwards this information to the company that developed the software for the smartphone app. Ten days after the patient has provided written informed consent, the practice nurse will inquire after the randomisation outcome and will inform the patient about it, and on how to use the paper patient diary. The patient receives an email with regard to the steps to be taken to receive the app-messages and on how to complete the online questionnaires. For the hospitals, the coordinating researcher will forward the randomisation outcome directly to the patient 10 days after the patient has provided written informed consent.

### The intervention

**Fig. 2:**
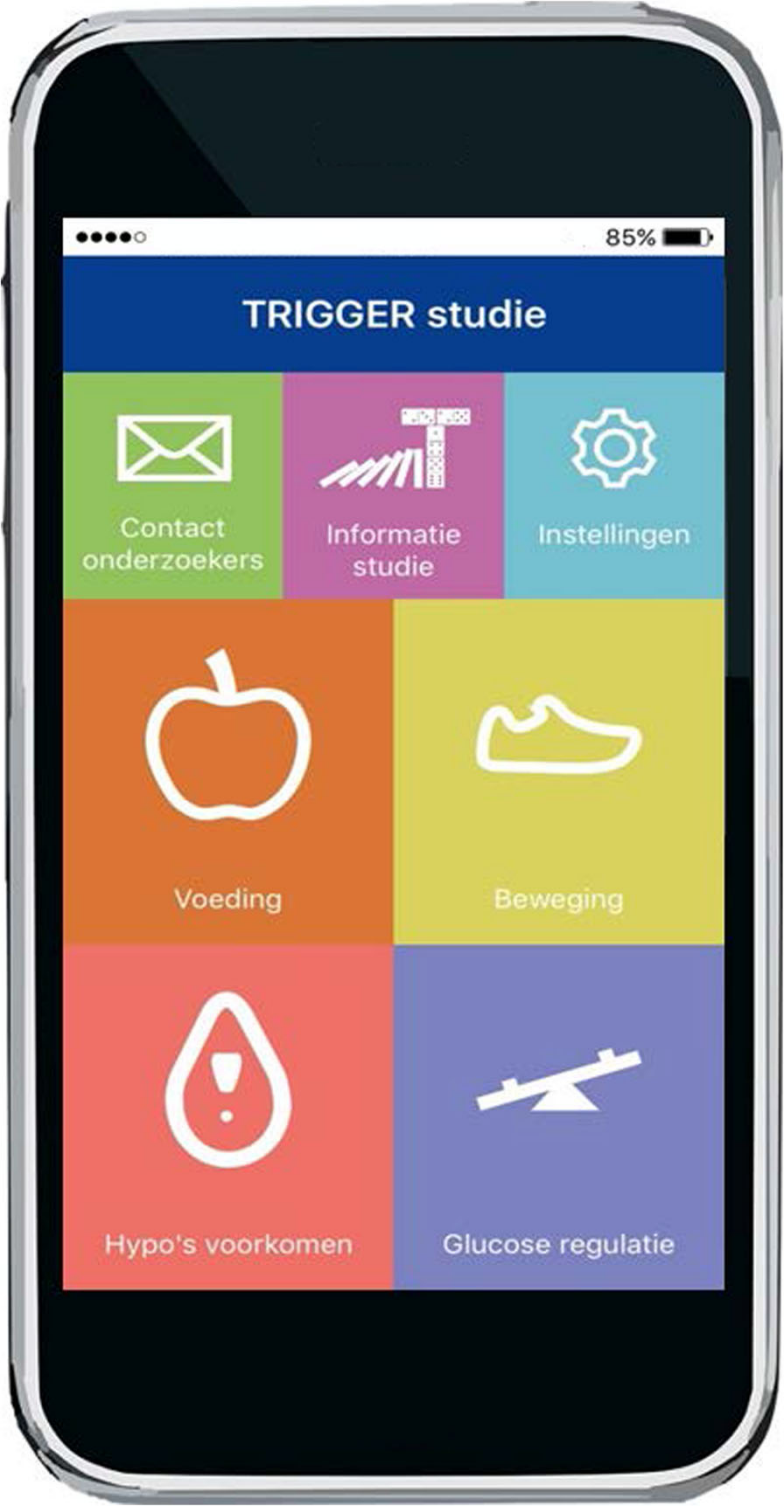
TRIGGER app home screen. Translation: contact onderzoekers = contact information; informatie studie = study information; instellingen = settings; voeding = nutrition; beweging = physical activity; hypo’s voorkomen = preventing hypoglycaemia; glucose regulatie = glucose regulation

Patients randomised to the intervention group will receive app-messages regarding dietary habits, physical activity, prevention of hypoglycaemia, and glucose variability (see Fig. 2). These app messages are clear, unidirectional messages containing specific goals, healthy living challenges, information, or questions (see Table 1 for examples). They are sent in Dutch only. The content of the intervention is determined by reviewing research literature of existing mHealth interventions and is in accordance with (inter-) national guidelines. An independent dietician, physiotherapist and practice nurse reviewed the messages’ content and wording. Afterwards, two T2DM patients on insulin reviewed the messages. Feedback was used for finalisation before the messages were entered into the app software system. Messages are framed grammatically correct, free of textese (“SMS language”), benefit-oriented, polite, nonaggressive and directive [28, 29]. Moreover, the intervention is tailored, according to patient’s preferences (see further). The intervention is free of charge, which is especially important for people with low socio-economic status; which might be a category of patients that needs self-management support but is not well approached by ‘conventional’ educational sessions [30].

**Table 1:** Examples or app messages

Category	Message
Dietary habits	- Fruit juices are not as healthy as many people think: they contain lots of sugar and calories. Try to consume fruit juices as infrequently as possible, eat fruit instead! - Don’t drink any soda today. Choose water, or tea or coffee without any sugar instead.
Physical activity	- Take the stairs instead of the elevator, wherever you are. Or take the elevator to one floor below and take the last stairs. All steps count! - People with a healthy weight are advised to be physically active for at least 30 min a day. People who are overweight are advised to have at least 60 min of physical activity each day.
Prevention of Hypoglycaemia	- Instruct your spouse, family, neighbour, colleague or a friend on how they should act when you have a hypoglycaemic event. - Do you have nocturnal hypoglycaemic events? Ask your doctor whether it is advisable to adjust your insulin dose.
Glucose variability	- Never inject insulin into the tough skin of insulin-induced lumps; because of slower absorption there, this may lead to blood glucose fluctuations. - When you have high blood glucose levels, it is important to drink a lot (1.5–2 l sugar free beverages per day) and to take your medication properly.

Users will receive a push notification to direct them to open the app when a new app-trigger is available. When the app is not opened in the following 24 h, a reminder SMS is sent: the SMS fall-back system. The number of reminder SMSs will be monitored.

The timing of the messages is either at random times between 9.30 a.m. and 8.00 p.m. or at times associated with the content of the message (e.g. messages containing advice for dinner are sent in the morning or early afternoon in order to get the receiver to adapt to that advice). The intervention will be tailored to the patients’ preferences, they can choose on:

- The topics: hypoglycaemia will be a mandatory topic, it should be combined with at least two of the following topics: dietary habits, physical activity or glucose regulation (including glycaemic variability).
- The frequency: two or six times per week, one app-trigger per day.
- The option to prolong the intervention with another 3 months after the first 6 months. During this period the patient will receive app-triggers with unaltered frequency and topics.

Apart from receiving app-triggers, the intervention group will receive usual care for their T2DM.

Patients randomised to the control group receive usual care according to the Dutch primary care guidelines, that advise two to four diabetes monitoring visits per year [2]. Through diabetes education from either the GP or the practice nurse, patients learn about target values for glycaemic control, lipids and blood pressure, a healthy lifestyle, recognising hyperglycaemic or hypoglycaemic episodes and how to respond to these episodes. Patients randomised to the control group are not able to use the smartphone app we developed. To increase the participation rate, patients in the control group will be given the opportunity to use the app after the study has ended.

### Technical details of the smartphone app

The smartphone app is compatible with Android and iOS operating systems. A proprietary personal health record (PHR) platform (“Gezondheidsmeter”, Curavista eHealth) is combined with both Android and iOS smartphone app technology for the end-user. The PHR platform is the host for the push message technology. The push message technology contains the algorithms for timely delivery, logging of end-user opening the message and fall-back SMS. Android and iOS technology is used to create an app for reception and storage (library) of the push messages. The mHealth intervention has no connection or interaction with any national or regional Health Information System. We did not involve end-users in usability testing but tested the technical aspects of the intervention ourselves. The PHR platform is subjected to Dutch privacy laws and has concluded a compliant data processing agreement. The messages do not contain any personal information. The logging data, such as successful delivery of messages, are anonymised by separating them from the personal data.

### Outcomes

The primary study outcome is glycaemic control, defined as the HbA1c level after a follow-up of 6 months in the app-trigger group compared to the control group. As a co-primary outcome we will analyse the percentage of patients who achieve an HbA1c level ≤ 53 mmol/mol (≤7%) without any hypoglycaemic event (plasma glucose < 3.5 mmol/L (< 63 mg/dL)). Secondary outcomes are BMI, body weight, waist circumference, insulin dose, lipid profile, blood pressure, number of hypoglycaemic events, glycaemic variability, self-management, food habits, physical activity, health status, diabetes-dependent quality of life, diabetes treatment satisfaction and satisfaction with the app (intervention group only) after 6 months. Moreover, we will investigate the cost-effectiveness of the intervention after 6 months, and the sustainability of the intervention effect. The latter is investigated in the intervention group only: we will compare the effect of 3 months prolonged use of the app to stopping with the use of the app, on HbA1c, on the percentage of patients who achieve an HbA1c level ≤ 53 mmol/mol (≤7%) without any hypoglycaemic event, BMI, and health status. Prolonged users are individuals from the intervention group who choose to continue the intervention after 6 months (T6); discontinued users are those who do not continue using the app after T6.

### Measurements

At baseline patients will complete seven questionnaires, regarding self-management activities, food habits, physical activity, health status, quality of life, diabetes dependent quality of life and diabetes treatment satisfaction. For a detailed description of the questionnaires see Table 2. Patients are asked to complete the baseline questionnaires before randomisation. After 3 months (T3) patients will complete two questionnaires; after 6 months (T6) patients will complete eight (control group) to nine (intervention group: extra questionnaire on satisfaction with the app) questionnaires (see Table 2). Patients in the intervention group also complete one questionnaire after 9 months of follow-up (T9) (see Table 2). All clinical variables are obtained by an online case report form (eCRF) completed by the practice nurse or diabetes nurse at T0, T6 and for the intervention group also at T9. In addition to anthropometric (length, weight, waist circumference and blood pressure) and laboratory parameters (HbA1c and lipid profile), the following demographic and clinical variables are collected: age, duration of diabetes, ethnicity, level of education, smoking status (current, former, never), presence of microvascular complications (nephropathy, retinopathy, neuropathy) and macrovascular complications (cardiovascular diseases), and diabetes medication use (including insulin injection frequency and dose).

**Table 2:** Description of questionnaires

Questionnaire	Description	Score range	Completed at
Summary of Diabetes Self-Care Activities Measure (SDSCA) [34]	11 Items assessing several aspects of the diabetes regimen: diet, exercise, blood glucose testing, foot care and smoking. Items measure how many days a patient has performed self-care activities in the last 7 days.	10 Items rated on an 8-point Likert scale, measuring how many days an activity is performed in the last week. One item measures smoking status (yes/no) and the amount of cigarettes smoked in the last week. Each of the domains is measured separately.	T0, T6
Food habits questionnaire (FFQ) [35]	18 Items assessing patient’s habits with regard to preparation of food, fatty food intake, dietary products and fruit and vegetable intake.	All items differ in the scoring ranges, varying from four scoring options (never, sometimes, frequently or always) to eight options (never to 7 days a week)	T0, T6
International Physical Activity Questionnaire (IPAQ) [36]	25 Items assessing how many days physical activities are performed during the past 7 days in four domains (work, transportation, housework and leisure-time), 2 items assess sedentary behaviour.	Total physical activity score is calculated as the sum of the number of minutes of total moderate activity for each subdomain, plus two times the number of minutes of total vigorous activity for each subdomain.	T0, T6
EQ-5D-5 L [37]	Quality of life questionnaire. The classification system (EQ-5D-Profile) covers mobility, self-care, daily activities, pain/discomfort and anxiety/depression. The Visual Analogue Scale (EQ-5D-VAS) is a graduated, vertical line, anchored at 0 (worst imaginable health state) and 100 (best imaginable health state).	For the EQ-5D-Profile each domain has five levels of functioning: from no problems to severe problems. For the EQ-5D-VAS the patient is asked to rate his/her health by marking a point on the EQ-5D-VAS that best reflects his/her actual health state.	T0, T3, T6, T9^a^
The Short Form (36) Health Survey (SF-36) [38, 39]	Generates a profile of scores on eight dimensions of health status: Physical Functioning (10 items), Role Physical (4 items), Bodily Pain (2 items), General Health (6 items), Vitality (4 items), Social Functioning (2 items), Role-Emotional (3 items) and Mental Health (5 items).	The different scales can be summarized in two component scores: the Physical Component Score and the Mental Component Score. Both scores range from 0 (least favorable health state) to 100 (most favorable health state).	T0, T6
The Audit of Diabetes-Dependent Quality of Life (ADDQoL) [40]	A measure of impact and importance of diabetes and its treatment on quality of life. The ADDQoL consists of 19 diabetes-specific items and two overview items. For each item the patient is asked how things would be without diabetes, with an impact rating and an importance rating.	The impact rating ranges from − 3 (very much better) to 1 (worse), the importance rating ranges from 3 (very important) to 0 (not at all important). The average weighted impact ADDQoL score is the sum of all the weighted impact scores in the nominator and of the number of domains in the denominator, and ranges from − 9 to 3.	T0, T6
Diabetes Treatment Satisfaction Questionnaire (DTSQ) [41]	Includes 8 items. In question 1 and 4–8 the satisfaction with the treatment is better if the scores are higher. In questions 2 and 3, lower scores indicate blood glucose levels closer to the ideal, and higher scores indicate problems.	Scores range per item from 6 (very satisfied) to 0 (very dissatisfied); total score range 36 to 0.	T0, T6
Satisfaction and usability of the app	Questionnaire newly developed by the researchers contains questions about the satisfaction with regard to receiving app-triggers, frequency, timing and comprehensibility, see Additional file 1.	All items will be rated on a 5-point Likert scale.	T6^a^
Outpatient clinic visits and paramedical health care use	Newly developed questionnaire contains one question on outpatient clinic visits (for patients recruited from primary care) or GP visits (for patients recruited from hospitals) and three questions on paramedical health care use: whether patients have visited a dietician, a physiotherapist or a specialised feet therapist the past 3 months.	All four questions are answered with yes or no. In case of a ‘yes’, patients have to fill in how many times they have visited the physician/therapist.	T3, T6

Abbreviations: *T0* baseline measurement, *T3* measurement after 3 months of follow-up, *T6* measurement after 6 months of follow-up, *T9* measurement after 9 months of follow-up

^a^Completed by intervention group only

**Fig. 3:**
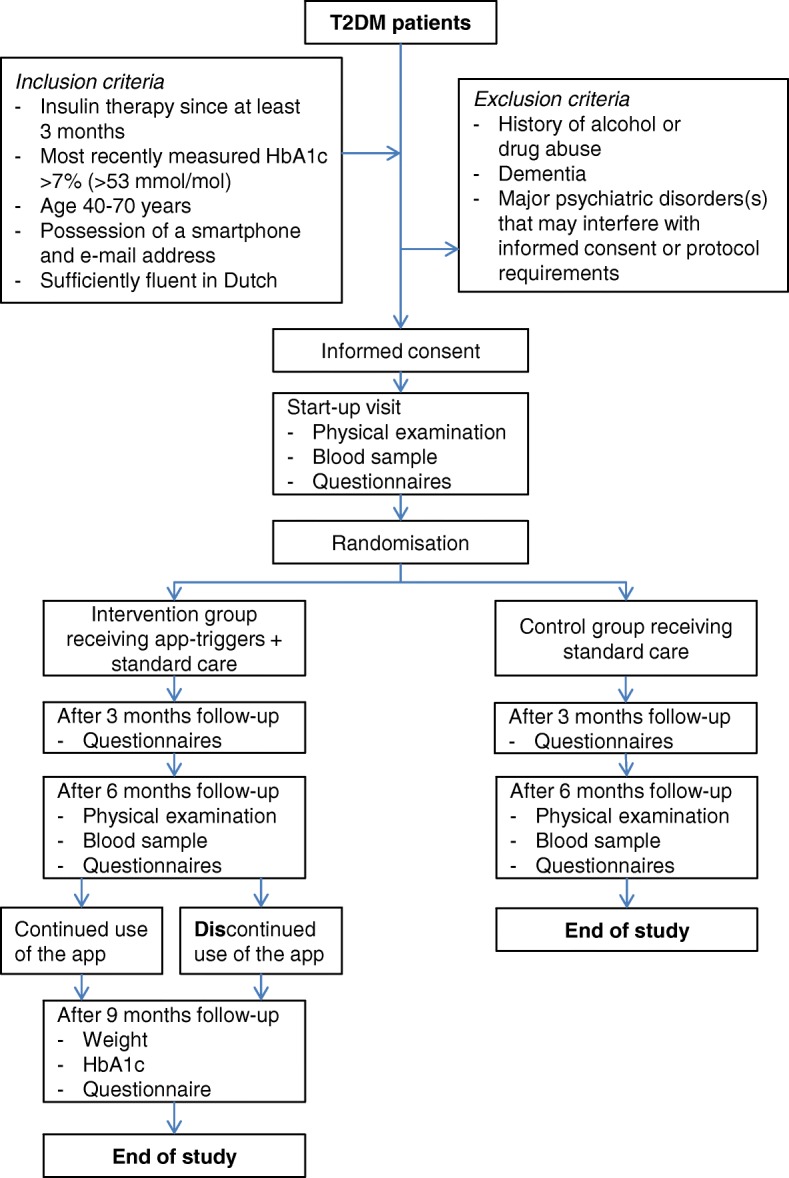
A schematic diagram with an overview of the study design and the main procedures

For the cost-effectiveness analysis, data on health care use are extracted from both the electronic medical record and from the questionnaires sent to the participants at T3 and T6. The number of hypoglycaemic events and the glycaemic variability are extracted at T6 (control group and intervention group) and at T9 (intervention group) from patient diaries, handed out to the patients at the start of the study. These diaries are designed for the purpose of this study only: patients record information on all hypoglycaemic events during the study, and once a week blood glucose values throughout the day (fasting plasma glucose and three pre-prandial values). For an overview of the study design and procedures: see Fig. 3.

### Monitoring, safety and confidentiality

A monitor will assess the safety and validity of the research data. Due to the low risk character of the intervention, only spontaneously reported adverse events will be reported in the result paper. Research data will be stored in code: all participants will receive their own serial number based on the study site and the eCRF number. Clinical data of the participants will be extracted from electronic medical records and anonymously provided on an eCRF to the coordinating research center.

### Concealment of allocation and blinding

The study is not blinded at patient level because of the nature of the intervention, which requires overt participation. The practice nurses/diabetes nurses/outcome assessors are also not blinded to patient allocation.

### Sample size

Based on the decrease in HbA1c in previous mHealth studies [9, 10, 18, 21, 31], we expect a mean difference in HbA1c level of 0.41% with a standard deviation of 1.1% between the intervention group and the control group after 6 months. To detect this difference in the primary outcome with an 80% power and a non-significant difference of 5% after 6 months, 228 T2DM patients (114 per treatment group) are needed. We used SAS version 9.4 (SAS Corp., Cary NC) for the sample size calculation.

### Analysis

Age and sex of our study population will be compared to those who declined to participate to investigate selection (bias). We will also compare baseline characteristics of our study population to those of similar Dutch T2DM patient cohorts.

Intention-to-treat analysis will be performed for the primary and secondary outcomes. If necessary, a multiple imputation technique will be used to handle missing data.

To analyse continuous variables we will use general linear models. Binary outcome variables will be analysed with logistic regression. To analyse the number of hypoglycaemic events we will use a Poisson regression. If necessary, the analysis will be adjusted for overdispersion or underdispersion.

Firstly, we will perform a univariable analysis to examine the intervention effect. Afterwards we will perform a multivariable analysis corrected for the following baseline characteristics: baseline value, age, sex, duration of diabetes and insulin dosage.

### Cost-effectiveness analysis

The cost-effectiveness of the intervention will be determined from a healthcare perspective. EQ-5D-5 L will be used to calculate quality-adjusted life-years (QALYs), following an area under the curve approach, with interpolation between measurements. All health care use will be valued following standard costs per unit as advocated for use in Dutch health economic evaluations. Cost differences between intervention and control groups will be related to QALY differences between groups. A probabilistic sensitivity analysis will be performed using bootstrapping. Results will be presented graphically in Cost-effectiveness Acceptability Curves.

## Discussion

Self-management is crucial for T2DM patients, especially for those on insulin therapy. However, to be effective, self-management education should be intensive [32], and thus will be costly. Moreover, worldwide there is a rapid increase in the number of T2DM patients. Against that background, cost-effective solutions to promote diabetes self-management are pivotal. If our newly developed app proves to be cost-effective, it could be used by all insulin treated T2DM patients because the messages are evidence-based and not region-specific. The topics ‘dietary habits’ and ‘physical activity’ can also be used to stimulate diabetes self-management in the non-insulin users. The app may be a standalone intervention but can also be used as part of a more comprehensive program. A possible limitation for delivery at scale might be the attitude of health care providers towards new technologies. Whereas the use of automated unidirectional messages may seem simple, a recently conducted systematic review and meta-analysis on this topic showed that one-way and two-way messages had a very similar effect, while two-way messages are usually more resource intensive [33].

An operational issue that may occur during the study is that patients stop reading the text messages, while they are not formally withdrawn from the study. However, since we are able to monitor the number of reminder SMSs sent (SMS fall back), we will be able to monitor the ‘adherence’. Moreover, this study is a real-life pragmatic trial; in real-life we also expect non-use or infrequent use of a smartphone app. Another issue that may arise, is that because all patients are informed about the study before they sign informed consent and before they are randomised, patients in the control group may also get aware of the existence of diabetes apps. Some patients in the control group might start using other diabetes apps during the study. To overcome this, patients in the control group will not have access to the smartphone app developed for the purpose of this trial, but will be offered to use it immediately after. Besides, both patients in the intervention and control group are equally able to use other diabetes apps.

Barriers to the adoption of the intervention among study participants may play a role. It is possible that this intervention is less effective among certain subpopulations that may be considered hard to reach (i.e. men, low socio-economic status and those who do not regularly attend health services). For that reason we will look at selection bias. This trial will include a relatively high number of T2DM patients, as suggested by a recent reviews [8, 19, 33]. It will add to the evidence base for mHealth interventions to stimulate self-management in insulin treated T2DM patients. The results may be of interest for health care providers, patients, patient organisations and policy makers who aim to increase diabetes self-management.

## Additional file

Additional file 1: Satisfaction and usability of the app questionnaire. (DOCX 18 kb)

## Abbreviations

ADDQoL: The Audit of Diabetes-Dependent Quality of Life
BMI: Body mass index
DTSQ: Diabetes Treatment Satisfaction Questionnaire
EQ-5D-5 L: EuroQol 5 Dimensions 5 Level instrument
FFQ: Food Frequency Questionnaire
GP: General practitioner
HbA1c: Glycosylated hemoglobin
IPAQ: International Physical Activity Questionnaire
mERA: mHealth Evidence reporting and assessment
mHealth: Mobile health
QALY: Quality-adjusted life-year
SDSCA: Summary of Diabetes Self-care Activities Questionnaire
SF36: The Short Form (36) Health Survey
SMS: Short message service
SPIRIT: Standard Protocol Items: Recommendations for Interventional Trials
T2DM: Type 2 diabetes mellitus
