# Supplementary material for: Effectiveness of diabetes self-management education via a smartphone application in insulin treated type 2 diabetes patients – design of a randomised controlled trial (‘TRIGGER study’)
Source: BMC Endocr Disord. 2018 Oct 22;18:74. doi: 10.1186/s12902-018-0304-9 (PMC6196442; doi:10.1186/s12902-018-0304-9)
Supplement: Supplementary file 1 — Satisfaction and usability of the app questionnaire. (DOCX 18 kb) [file 12902_2018_304_MOESM1_ESM.docx]

**Additional file 1**. Satisfaction and usability of the app questionnaire

The following questions concern the phone application you have been using during the past months. Please answer the questions by colouring the appropriate box underneath each question.

1. Receiving the messages motivated me to live healthily

Completely disagree ○ ○ ○ ○ ○ Completely agree

2. Receiving the messages taught me a lot about diabetes

Completely disagree ○ ○ ○ ○ ○ Completely agree

3. The messages were sent at the right moments

Completely disagree ○ ○ ○ ○ ○ Completely agree

4. The information in the messages was difficult to understand

Completely disagree ○ ○ ○ ○ ○ Completely agree

5. If I were to proceed with the programme I would want to receive fewer messages

Completely disagree ○ ○ ○ ○ ○ Completely agree

6. I would recommend this application to family and friends with diabetes

Completely disagree ○ ○ ○ ○ ○ Completely agree

7. The messages prompted me to improve the healthiness of my diet

Completely disagree ○ ○ ○ ○ ○ Completely agree

8. The messages encouraged me to exercise more

Completely disagree ○ ○ ○ ○ ○ Completely agree

9. Because of the messages, I am more capable of dealing with hypoglycemia
Completely disagree ○ ○ ○ ○ ○ Completely agree

10. The messages were applicable to me
Completely disagree ○ ○ ○ ○ ○ Completely agree

11. I often made use of the messages (for example, I took the stairs or cooked a healthy meal)

Completely disagree ○ ○ ○ ○ ○ Completely agree

12. I often forgot to make use of the messages

Completely disagree ○ ○ ○ ○ ○ Completely agree
